# Visualizing data: Trends in smoking tobacco prices and taxes in India

**DOI:** 10.12688/gatesopenres.12894.1

**Published:** 2019-01-17

**Authors:** G. Emmanuel Guindon, Tooba Fatima, David X. Li, Alexandra Joukova, Jitender Sudhir, Sujata Mishra, Frank J. Chaloupka, Prabhat Jha

**Affiliations:** 1 Centre for Health Economics and Policy Analysis, McMaster University, Hamilton, Ontario, L8S 4K1, Canada; 2 Department of Health Research Methods, Evidence, and Impact, McMaster University, Hamilton, Ontario, L8S 4K1, Canada; 3 Department of Economics, McMaster University, Hamilton, Ontario, L8S 4K1, Canada; 4 Department of Medicine, University of California, Los Angeles, Los Angeles, California, 90095, USA; 5 Centre for Global Health Research, St Michael's Hospital, Toronto, Ontario, M5B 1W8, Canada; 6 Dalla Lana School of Public Health, University of Toronto, Toronto, Ontario, M5T 3M7, Canada; 7 Division of Health Policy and Administration, School of Public Health, University of Illinois at Chicago, Chicago, Illinois, 60612, USA; 8 National Bureau of Economic Research, Cambridge, Massachusetts, 02138, USA

**Keywords:** smoking, tobacco, cigarette, bidi, India, price, tax, data visualization

## Abstract

**Background** **:** Tobacco smoking remains a leading risk factor for disease burden globally. In India alone, about 1 million deaths are caused annually by smoking. Although increasing tobacco prices has consistently been found to be the most effective intervention to reduce tobacco use, the documentation of prices and taxes across time and space has not been an essential component of tobacco control surveillance in most jurisdictions. This study aimed to examine, using graphical methods, trends in smoking tobacco taxes and prices in India at national and state-level.

**Methods** **:** We used retail prices, price indices, and unit values (household expenditures on a commodity divided by the quantity purchased) collected and reported by government agencies. For bidis and cigarettes, we examined current and real (inflation-adjusted) prices, affordability (cost in terms of income), and key tax changes at both national and state-level.

**Results** **:** We show that real prices of bidis and cigarettes were relatively flat (even decreasing in the case of bidis) between 2000 and 2007, and clearly increasing from 2010. When rising income is taken into account, however, both cigarettes and bidis have become more affordable since 2000. We found that some but not all tax changes were accompanied by price changes and in particular, that tax decreases did not result in price decreases.

**Conclusion** **:** It is feasible to evaluate tax and price policies at national and regional level using routinely collected data.

## Introduction

For more than three decades, tobacco smoking has remained a leading risk factor for premature mortality globally ^1^,^2^. While tobacco-attributable deaths are predicted to decline in high-income countries, they are predicted to double from 3.4 million to 6.8 million in low- and middle-income countries ^3^. In India, despite modest decreases in the prevalence of tobacco smoking (i.e., bidis ^i^ and cigarettes), the number of male smokers aged 15–69 years has increased substantially over the last 15 years, with a current population of more than 100 million adult smokers ^4^. About 1 million Indians are killed by smoking per year, most of these occurring at ages 30–69 years, where decades of good life are lost compared to otherwise similar non-smokers ^5^. Unlike most countries, the most common type of smoking tobacco product in India are bidis, followed by cigarettes. Over the last decade or so, cigarettes, however, have started displacing bidis, particularly among young adult and poorer men ^4^.

In India, the power to levy ‘duties of excise on tobacco’ lies with the central (i.e., federal) government ^6^. The central government imposes a number of taxes on tobacco products — duties in the form of central excise on the sale of different tobacco products, a surcharge towards the National Calamity Contingency Fund, and special excise duties. The India central tobacco tax structure is overly complex, even chaotic ^7^. The basic excise duty (BED), by far the most important tax imposed on cigarettes, varies by length and whether or not cigarettes are filtered. In June 2018, the specific cigarette tax on the most popular filter cigarettes (> 60 to 70 mm) was approximately 28 Rupees (Rs) per pack of 10 cigarettes, about USD 0.40 or € 0.35. Taxes on bidis, however, are negligible. From the mid-2000s, States and Union Territories began switching away from a system based on numerous sales taxes to one more focused on value-added taxes (VAT) ^8^. By 2008, most States and Union Territories had introduced VAT on goods, including bidis and cigarettes. State VAT rates on bidis and cigarettes have varied widely through time, between States, and between the products themselves (bidis and cigarettes). In July 2017, all State VATs were repealed and replaced by a national Goods and Services Tax (GST) that uses four tax rates: bidis and cigarettes are taxed at 28%, the highest rate. Cigarettes that are no more than 75 mm in length face an additional 5% while cigarettes that are greater than 75 mm are taxed an additional 36% ^9^.

Increasing tobacco prices has repeatedly been found to be the most effective intervention to curb tobacco use. Moreover, in high-income countries, youth as well as individuals of lower socioeconomic status have been found to be generally more responsive to changes in prices ^10^,^12^. Given the importance of price and tax measures to reduce tobacco use, keeping track of prices and taxes across time and space ought to be an essential component of tobacco control surveillance. It is, however, a component that is too often ignored. Existing studies that examined trends in cigarette prices and affordability have almost exclusively relied on Economist Intelligence Unit (EIU) city-level price data ^13^,^15^. The EIU data are collected only semi-annually and cover at best a handful of cities. Most recently, the EIU collected data from just four major Indian cities (Bangalore, Chennai, Mumbai, New Delhi), and from only two (Mumbai, New Delhi) in the early 2000s. More recently, a few studies have made use of self-reported data. For example, Kostova *et al*. ^16^. examined cross-sectional self-reported data from fifteen countries including India. Such an approach had the advantage of allowing the examination of prices paid by household- or individual-level characteristics but provided no temporal information. The Framework Convention on Tobacco Control (FCTC) recognizes the importance of prices and taxes and recommends monitoring (Articles 6 and 20) ^17^. Unfortunately, the current reporting of prices is very limited and poorly documented. Additionally, numerous errors in WHO’s FCTC implementation database have been documented ^18^. Given that retail price data are collected regularly by government agencies such as national statistics offices, the failure to track and use these data is of concern. Given India’s size and variations across states in smoking rates, income and income distribution, culture, and religion, city-level data fail to capture important spatial variations. Similarly, EIU city-level data are only measured twice yearly which makes it difficult to look at the effects of taxes on prices and subsequently, the effects of taxes and prices on tobacco use.

Recently, there has been calls for economists and public policy practitioners to make better use of data visualization ^19^,^20^. Our objective is to examine, using graphical methods, trends in smoking tobacco taxes and prices in India at national and state-level.

## Methods

India has a number of price indices: consumer price index (CPI) for Industrial Workers, CPI-IW; CPI for Agricultural Labourers and Rural Labourers, CPI-AL/RL; CPI for Urban Non-Manual Employees, CPI- UNME (discontinued in 2011 and replaced by a rural/urban CPI); and, a Wholesale Price Index (WPI). All-India all-items price indices are publicly available through various government online resources. All-India price indices for bidis and cigarettes are available online for some, but not all, indices. The Office of the Economic Adviser, Ministry of Commerce & Industry publishes monthly bidi and cigarette wholesale price indices while the Labour Bureau, Ministry of Labour and Employment publishes bidi and cigarette retail price indices, based on CPI-IW ^ii^. Village and centre-level prices are available from the Labour Bureau in paper and electronic format. We compiled a unique set of monthly data, covering three price indices over more than 15 years, which involved the digitization of more than 12 000 pages (most pre-2006 village and centre-level data were available in paper format only). We interpolated missing price data using piecewise cubic Hermite interpolation at village or centre-level ^21^.

*Consumer Price Index, Industrial Workers (CPI-IW).* Compiled and collected by the Labour Bureau of the Ministry of Labour and Employment, the objective of CPI-IW is to measure price changes for goods and services consumed by workers in seven industrial sectors (factories, mines, plantations, railways, public motor transport undertakings, electricity generation and distribution establishments, and ports and docks). Prices for about 370 items (including bidis and cigarettes) are collected monthly from about 300 price collection markets across about 80 centres (from most, but not all, states). CPI-IW is used primarily to determine the dearness allowance being paid to Central/State government and industrial sector employees based on revision and fixation of minimum wages. We have obtained CPI-IW retail prices for bidis and cigarettes at centre-level from January 1998 to March 2018 for most, but not all, months and centres. We have also obtained national indices for bidis and cigarettes from January 1998 to April 2018.

*Consumer Price Index, Agricultural Labourers and Rural Labourers (CPI-AL/RL).* Compiled by the Labour Bureau of the Ministry of Labour and Employment and collected monthly by the National Sample Survey Office (NSSO) from about 1500 markets in 600 villages in 20 states, CPI-AL/RL has the objective of measuring price changes for goods and services consumed by agricultural and rural labourers. CPI-AL/RL is used to guide the revision of minimum wages of agricultural and rural workers. We have obtained CPI-AL/RL retail prices for bidis and cigarettes at village-level from January 1998 to April 2014 for most, but not all, months and villages.

*Wholesale Price Index (WPI).* Compiled by the Office of the Economic Adviser, Department of Industrial Policy & Promotion, Ministry of Commerce & Industry, WPI measures weekly price movement, at the level of either the wholesaler or the producer. Prior to the 2011–12 revision, wholesale prices represented ex-factory/ex-mining prices of commodities minus trade discount (if any) plus central excise duty (including cess) and do not take into account retail margins. WPI calculated with 2011–12 base year no longer include taxes. Wholesale prices are collected for more than 600 commodities including bidis and cigarettes. The overall monthly all-India WPI is available online from April 1953; a composite of bidi, cigarettes, tobacco and zarda ^iii^ is available from 1971; and, disaggregated price indices for bidis and cigarettes are available from April 1982.

*Self-reported unit values.* Self-reported prices allow the examination of prices by smokers’ characteristics (e.g., socioeconomic status). Akin to most household surveys, India’s National Sample Survey (NSS) ^iv^ collects, at household level, expenditures and quantity consumed for various items such as food, tobacco, and alcohol products. Self-reported expenditures and unit value (average expenditure per unit) can then be used as a proxy for price. We used data from National Sample Surveys conducted between 1999-00 and 2011-12 (NSS 55-57, 59-64, 66, 68) ^v^. We inspected unit values for outliers. First, we charted box plots and histograms. Second, we removed unit values whose logarithms lied more than 2.5 standard deviations from the mean ^22^.

*Affordability.* We explored affordability (i.e., cost in terms of income) using both retail prices and self-reported prices. First, we used quarterly growth rates of real gross domestic products (GDP) from the Quarterly National Accounts (growth rate compared to previous quarter, seasonally adjusted) ^vi^. To weigh GDP by population, we used annual population estimates (≥ 15 years old) from the United Nations Population Division ^vii^. We converted annual population estimates to quarterly estimates using the proportional Denton method ^23^. Second, we used NSS data to construct a measure of affordability at household level that represents the cost of purchasing 100 packs of 10 cigarettes or bundles of 25 bidis as a proportion of total monthly household expenditures.

*Taxation.* We compiled relevant tax rates at both central and state-level from a large number of government (most often tax schedules from finance departments) and media reports.

*Data handling and visualization:* For figures that present state-level CPI data, in addition to average prices, we show for each data point, the minimum and maximum prices (top panel), 95% confidence intervals ^viii^ (middle panel) and sample sizes (bottom panel). Data points in dark blue represent average prices that did not require any interpolations. Data points in light blue represent average prices that were calculated from prices that were all interpolated. Data points in ‘mid-blue’ represent average prices that were based on at least one interpolated price. All data analyses and graphics were done using Stata/MP 15.1.

## Results

We present graphically trends in current and real (i.e., prices adjusted for overall inflation) prices of bidis and cigarettes. Whenever relevant, we superimposed key tax changes; central total cigarette taxes are presented in Table 1.

**Table 1.** Total central excise duty rates on cigarettes, Rs per 1000 cigarettes (current Rs).

	Non-filter		Filter				
Year	≤ 60 mm	> 60–70 mm	≤70 mm		> 70–75 mm	> 75–85 mm	> 85 mm
1993/94	120	250	330		630	850	
1994/95	60	280	370		710	950	1350
1995/96	60	300	400		750	1000	1350
1996/97	75	315	430		800	1070	1350
1997/98	90	350	500		820	1100	1470
1998/99	100	370	550		900	1200	1470
1999/00	110	370	550		945	1200	1545
2000/01	115	390	580		1090	1260	1780
2001/02	135	450	670		1090	1450	1780
2002/03	135	450	670		1090	1450	1780
2003/04	135	450	670		1090	1450	1780
2004/05	135	450	670		1090	1450	1780
2005/06	150	495	740		1200	1595	1960
2006/07	160	520	780		1260	1675	2060
2007/08	168	546	819		1323	1759	2163
2008/09	819	1323	819		1323	1759	2163
2009/10	819	1323	819		1323	1759	2163
	…	…	≤60 mm	> 60–70 mm	…	…	…
2010/11	669	1473	669	969	1473	1959	2363
2011/12	669	1473	669	969	1473	1959	2363
	≤ 65 mm	> 65–70 mm	≤65 mm	> 65–70 mm	…	…	…
2012/13	669	1473	669	969	1473	1959	2363
2013/14	669	2027	669	1409	2027	2725	3290
2014/15	1150	2250	1150	1650	2250	3290	
2015/16	1440	2590	1440	1900	2590	3790	
2016/17	1585	2850	1585	2090	2850	4170	
2017 *	1681	3021	1681	2216	3021	4421	
2017/18 **	2166	3813	2166	2837	3813	4405	

Note. * = March–July 2017; ** = From 18 July 2017; An additional tax of 5% is appplied to all cigarettes < 75 mm; and 36% for cigarettes ≥ 75 mm.

**Figure 1.**
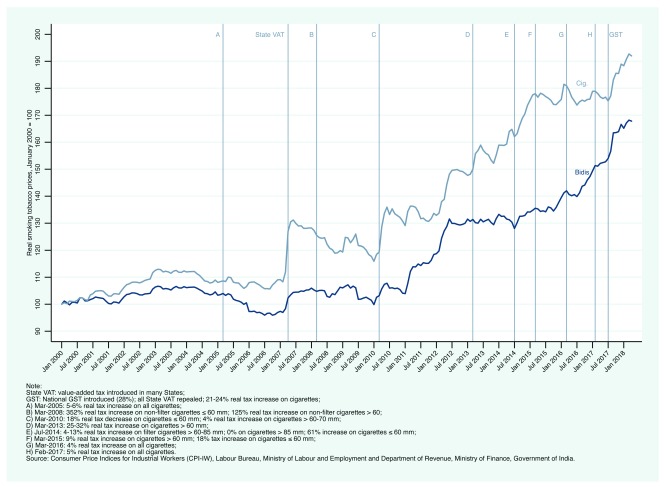
Trends in real prices in India: Consumer Price Indices for Industrial Workers for bidis, cigarettes and all-items, January 2000 - April 2018.

**Figure 2.**
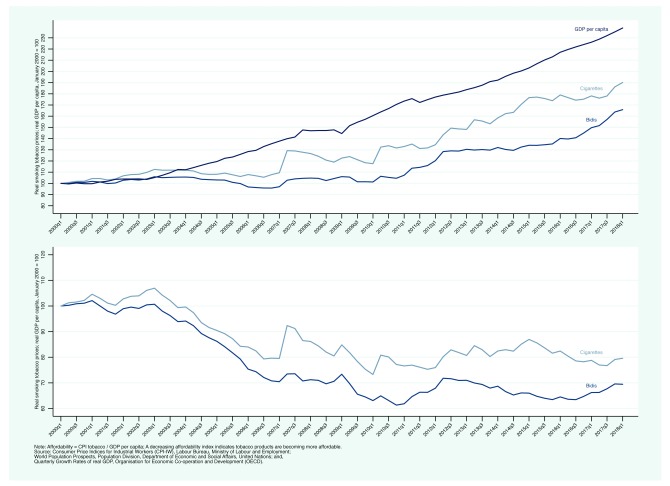
Trends in affordability in India: Consumer Price Indices for Industrial Workers for bidis and cigarettes, Quarterly Growth Rates of real GDP, January-March 2000 — January-March 2018.

First, we present national trends in nominal and real prices and affordability. Figure 1 presents national CPI-IW real price data from January 2000 to April 2018 for bidis, and cigarettes. ( Figure A1 (Extended data ^24^.) presents the same data in nominal terms). Real cigarette prices increased nearly two-fold between 2000 and 2017 while real bidi prices increased by more than 160%. Put differently, between 2000 and 2017, cigarette and bidi real prices increased at an annual rate of about 3 and 3.7%, respectively. These price increases may appear substantial but do not take into account changes in income over the same period. Figure 2 presents a measure of affordability. The top panel presents nominal indices for bidis and cigarettes and GDP per capita; the bottom panel presents our measure of affordability, CPI tobacco / GDP per capita (a decreasing affordability index indicates that tobacco products have become more affordable). Figure 2 shows clearly that income growth has outpaced the increases in bidi and cigarette prices. By early 2018, bidis and cigarettes were about 30 and 20% more affordable than they were in early 2000, respectively. Figure A2– Figure A3 (Extended data ^24^) present similar trends based on CPI-AL/RL while Figure A4– Figure A5 present WPI data. The diverging trends in later years between WPI and CPI-IW are due to the change in WPI methodology (WPI calculated with 2011-12 base year no longer include taxes).

A closer look at tax policy changes highlighted in Figure 1 clearly indicates that some policy changes affected average prices, and some did not. For example, in March 2010, although real tax rates decreased for most cigarette categories, taxes on the most popular cigarettes (> 60 to 70 mm) increased by 18% (4.4% after adjusting for inflation). The effect on cigarette prices seems evident. Relatively large tax increases in 2013 and 2014 also seem to have pushed cigarette prices upward. Subsequent tax changes in 2015, 2016, and 2017 seemed to have had no effect on cigarette prices, until the tax overhaul in July 2017. By year end, bidi and cigarette prices had increased by 6.5 and 5.8% in excess of overall inflation.

Second, we present state-level CPI prices. Figure 3ab and Figure 4ab show the evolution of bidi and cigarette prices and state-level smoking tobacco taxation in Rajasthan and Uttar Pradesh, between January 1998 and March 2018. From 2007, Rajasthan aggressively increased its VAT on bidis and cigarettes. Bidis and cigarettes were first taxed at a rate of 12.5% in April 2007. By April 2013, the rate had been subsequently increased to 20, 40, 50, and 60%. Over the same period, bidi prices (per bundle of 25) increased from less than 5 Rs to between 12 and 14 Rs ( Figure 3a). Similarly, cigarette prices increased from less than 20 Rs (per pack of 10) to more than 45 Rs ( Figure 3b). In contrast to Rajasthan, Uttar Pradesh did not apply its VAT to bidis. VAT was first applied on cigarettes in January 2008 at a rate of 4%. By 2012, the VAT rate on cigarettes had reached 50% but in May 2013, the rate was reduced in half to 25% (in contrast to Rajasthan that continued to increase its VAT rate). Uttar Pradesh then reversed course in 2015 with an increase to 40%. Bidi prices increased from about 2 Rs per bundle in 2000 to between 3 and 8 Rs in 2014 ( Figure 4a). Comparing CPI-IW and CPI-AL/RL data suggests that bidi prices may have been substantially lower in rural areas than in urban areas in Uttar Pradesh. Nominal cigarette prices increased steadily between 2006 from just under 15 Rs to just over 35 Rs per pack ( Figure 4b). Of note is the continuing trend after the 50% decrease in VAT which suggest that manufacturers simply increased their profit margins at the expense of government tax revenue. Figure A6– Figure A8 (Extended data ^24^) present similar data for Andhra Pradesh, Kerala, and Maharashtra.

**Figure 3a.**
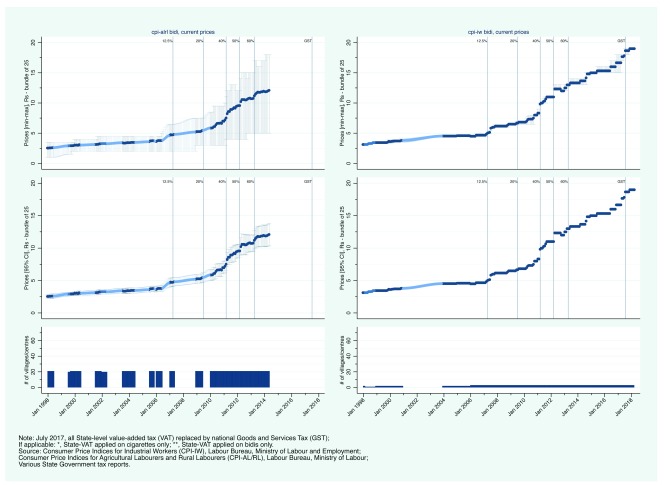
Trends in current bidi prices and smoking tobacco taxation in Rajasthan, January 1998 - March 2018.

**Figure 3b.**
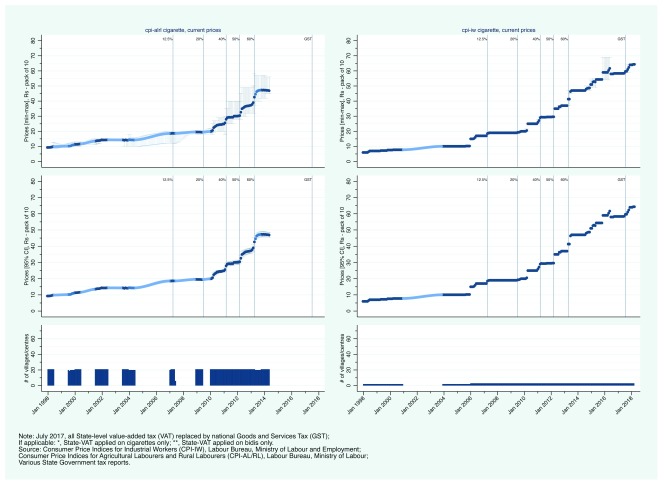
Trends in current cigarette prices and smoking tobacco taxation in Rajasthan, January 1998 - March 2018.

**Figure 4a.**
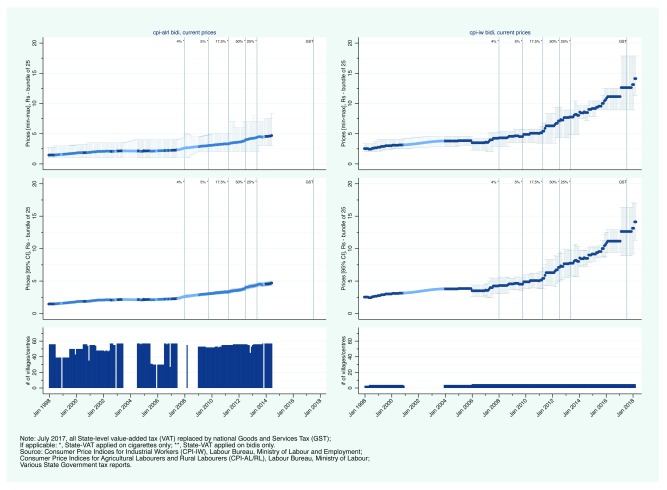
Trends in current bidi prices and smoking tobacco taxation in Uttar Pradesh, January 1998 - March 2018.

**Figure 4b.**
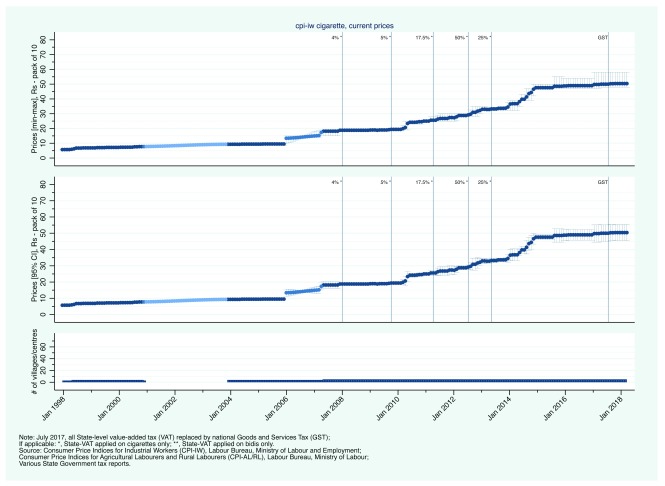
Trends in current cigarette prices and smoking tobacco taxation in Uttar Pradesh, January 1998 - March 2018.

Third, we present prices paid by household of differing SES-levels and between rural and urban households based on NSS unit values. Figure 5a and Figure 5b present trends in current bidi and cigarette prices (top panel) and affordability (middle panel) by household total expenditures tertiles (in the top and middle panels, data points in dark, ‘mid’, and light blue represent self-reported prices paid by low-, mid-, and high-SES household, based on household total expenditure, respectively; the bottom panel reports the total number of households that reported both quantity purchased and expenditure). As expected, low-SES households reported paying lower prices than high-SES households. Of note is the increasing gap between high- and low-SES households in self-reported bidi prices. In the early 2000s, differences were negligible. In the early 2010s, low-SES household reported paying nearly 2 Rs less per bundle of 25 bidis. In contrast, the gap between high- and low-SES households in self-reported cigarette prices remained more or less the same between 2000 and 2012. In contrast to rising current prices, the affordability of bidis and cigarettes remained relatively constant. Figure A9a and b (Extended data ^24^) present the data by rural/urban status. There were small differences in self-reported unit values for bidis between rural and urban households while urban households reported slightly higher unit values for cigarettes, with no discernible change through time.

**Figure 5a.**
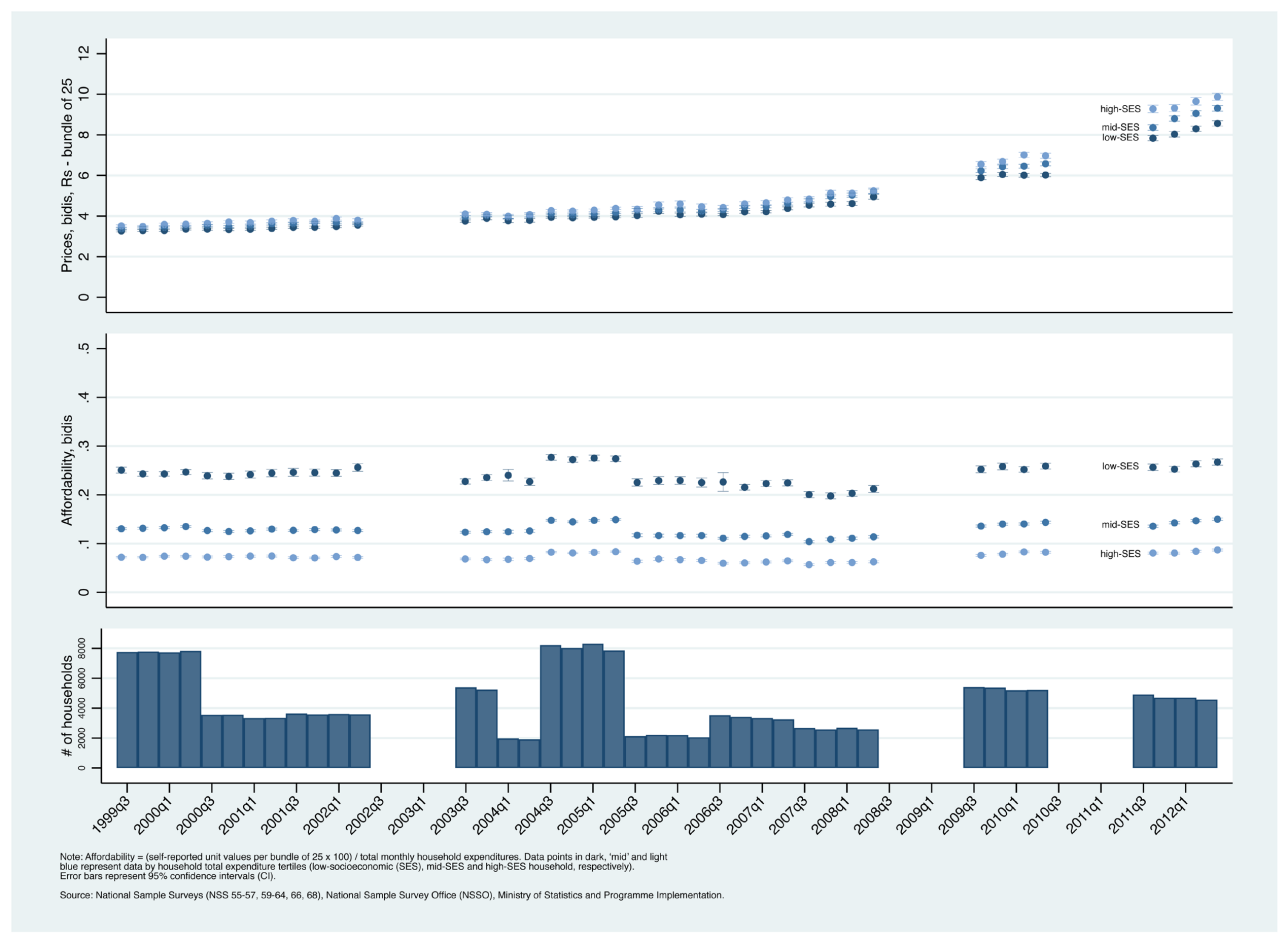
Trends in current bidi prices and affordability by socioeconomic status in India: National Sample Surveys, July-September 1999 — April-June 2012.

**Figure 5b.**
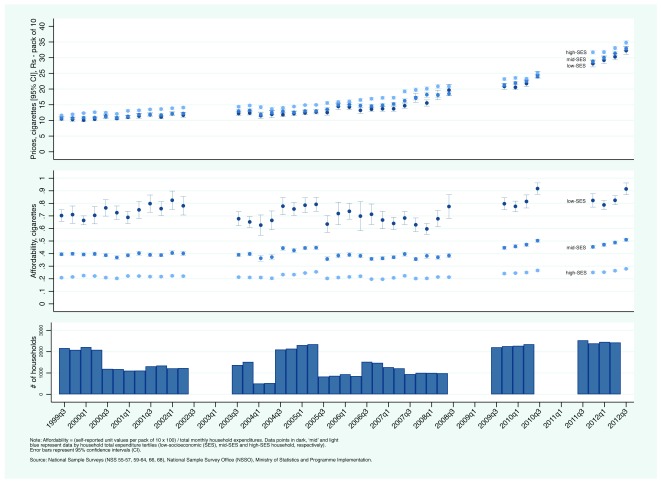
Trends in current cigarette prices and affordability by socioeconomic status in India: National Sample Surveys, July-September 1999 — April- June 2012.

## Discussion

*Main findings.* We presented data that clearly show that average nominal prices of bidis and cigarettes in India have increased (at a relatively increasing rate) since January 2000. In real terms (i.e., after adjusting for overall inflation), prices of bidis and cigarettes were relatively flat (even decreasing in the case of bidis) between 2000 and 2007 and clearly increasing from 2010. The aforementioned trends, however, do not take into account increasing income, which increased on average by almost 6% per year. When rising income is taken into account, both cigarettes and bidis have not become less affordable between 2000 and 2018. We also found that some, but not all, tax changes were accompanied by price changes. The extent to which tobacco manufacturers adjusted prices following tax changes varied in time as well as by states and products.

*Strengths.* We used two approaches to examine bidi and cigarette prices at national and state level. First, we used retail prices collected by the Labour Bureau. Second, we used unit values reported by households in 11 waves of the National Sample Survey. This approach provided both ample time and spatial variations. We dealt with missing values by first carefully examining and removing outliers and second, by interpolating missing values using piecewise cubic Hermite interpolation at the lowest level (i.e., village and centre-level). We constructed a dataset of bidi and cigarette prices and tax policies at national and state-level from 1998. The data we present came from an array of data sources and varied both in time and space which makes it difficult to present in tabular format. We show that graphical methods can be used to present data more effectively. For example, in some of our figures, we presented secular trends in monthly average prices, along with minimum and maximum prices, 95% confidence intervals, and sample sizes. We then superimposed key tax changes and indicated which monthly data point had been interpolated.

*Limitations*. For some of the state-level trends based on CPI-IW and CPI-AR/RL data, there were obvious breaks in the price series in January 2006 due, in part, to the addition of new markets being sampled or a change in the products sampled. Consequently, any changes that occurred between December 2005 and January 2006 should be treated with caution. Although price and unit value data are available for smokeless tobacco, the product diversity makes it hard to makes sense of the data. For example, price and unit value data were, most often than not, bimodal.

*Implications for policy, practices and research*. Although bidi and cigarette real prices have increased substantially between 2000 and 2018, bidis and cigarettes were nevertheless about 40 and 20% more affordable than they were in early 2000, respectively. Given that the International Monetary Fund (IMF) projects India’s GDP per capita to grow by about 9 to 11% annually, over the next five years, large and sustained tax increases will be required to prevent bidis and cigarettes from becoming yet more affordable ^25^. The data presented show that some but not all tax changes were accompanied by price changes. Of importance is the observation that tax *decreases* did not result in *price* decreases. This is unsurprising as cigarette manufacturers typically protest any tax increases (even benign ones) and often fully pass-through or even overshift tax increases ^26^,^29^.

The main implication for policy is the need for much larger tax increases that are implemented quickly, and are far above the rate of income growth. Large increases in excise taxation have the benefit of also signaling to smokers that future price increases are likely. Moreover, use of excise taxation to narrow the gap between the least and most expensive lengths is needed to decrease downward substitution, as well as to capture a greater proportion of the revenue ^30^,^31^. Indeed, the relatively small increases in excise taxation in India have created opportunities for the tobacco industry to raise their profits.

The dataset we have compiled demonstrate the feasibility of documenting prices and taxes at national, state, and district-level by making use of routinely collected data to evaluate current tax and price policies. Such data can and should be routinely compiled and examined by tobacco control practitioners in India and elsewhere, as nearly all countries collect monthly price data to construct price indices such as consumer price indices. Moreover, these price data can be linked with existing surveys such as the National Sample Surveys and improve the assessment of the impact of price changes on tobacco use ^32^,^36^.

In sum, we establish the feasibility of using routinely collected tax and price data from national consumer price surveys to evaluate tobacco control policies. The CPIs are collected widely and offer a low-cost, generally publicly available dataset to track tobacco taxation. In India, these routine data reveal that the sub-optimal use of large increases in excise taxes are not changing affordability of cigarettes in particular ^7^.

## Data availability

The data required to construct all figures that present price indices are publicly available from various Indian government sources and the OECD ( Figure 1– Figure 4, Figure A1– Figure A8).

We have deposited the data that were used to create these figures along with our Stata codes in an online repository - Open Science Framework:

OSF: Dataset 1. Visualizing data: Trends in smoking tobacco prices and taxes in India https://doi.org/10.17605/OSF.IO/UJXD6 ^24^

License: CC0 1.0 Universal

- Figures 1, A1-A5: all_india_m.csv; graphics_all-india_vDec2018_1.do
- Figure 2: all_india_q.csv; graphics_all-india_vDec2018_1.do
- Figures 3-4, A6-A8: cpi_iw_Dec2018_1.csv; cpi_al-rl_vDec2018_1.csv; cpi_iw_centre_codes.dta; cpi_al-rl_village_codes.dta; graphics_vDec2018_1-iw_alrl.do

The data required to create Figures 5 and A9 are not publicly available but can be obtained from the the Ministry of Statistics and Programme Implementation, Government of India for a fee (see http://mospi.nic.in/sample-surveys for more details).

- Figures 5, A9: graphics_vDec2018_1-nss_uv.do; graphics_vDec2018_1-nss_aff.do

### Extended data

The following supplementary figures are available from OSF

OSF: Extended data. Visualizing data: Trends in smoking tobacco prices and taxes in India https://doi.org/10.17605/OSF.IO/UJXD6 ^24^

License: CC0 1.0 Universal

- Figure A1. Trends in current and real prices in India: Consumer Price Indices for Industrial Workers for bidis, cigarettes and all-items, January 2000 - April 2018.
- Figure A2. Trends in current prices in India: Consumer Price Indices for Agricultural Labourers and Rural Labourers for bidis, cigarettes and all-items, January 2000 ‐ April 2014.
- Figure A3. Trends in real prices in India: Consumer Price Indices for Agricultural Labourers and Rural Labourers for bidis and cigarettes, January 2000 ‐ April 2014.
- Figure A4. Trends in current prices in India: Wholesale Price Indices for bidis, cigarettes and all-items, January 2000 — April 2018.
- Figure A5. Trends in real prices in India: Wholesale Price Indices for bidis and cigarettes, January 2000 — May 2018.
- Figure A6a. Trends in current bidi prices and smoking tobacco taxation in Andhra Pradesh, January 1998 ‐ March 2018.
- Figure A6b. Trends in current cigarette prices and smoking tobacco taxation in Andhra Pradesh, January 1998 ‐ March 2018.
- Figure A7a. Trends in current bidi prices and smoking tobacco taxation in Kerala, January 1998 ‐ March 2018.
- Figure A7b. Trends in current cigarette prices and smoking tobacco taxation in Kerala, January 1998 ‐ March 2018.
- Figure A8a. Trends in current bidi prices and smoking tobacco taxation in Maharashtra, January 1998 ‐ March 2018.
- Figure A8b. Trends in current cigarette prices and smoking tobacco taxation in Maharashtra, January 1998 ‐ March 2018.
- Figure A9a. Trends in current bidi prices by rural/urban status in India: National Sample Surveys, July-September 1999 — April-June 2012.
- Figure A9b. Trends in current cigarette prices by rural/urban status in India: National Sample Surveys, July-September 1999 — April-June 2012. 

## Footnotes

Also known as beedis, beeris or biris, bidis are slim, hand-rolled, unfiltered cigarettes rolled in a tendu leaf or temburni leaf and held together by a cotton thread that contain about 1⁄4 the tobacco of cigarettes. Bidis are available branded and unbranded and are about four to eight cm in length.

WPI, eaindustry.nic.in; CPI-IW, labourbureaunew.gov.in.

Zarda is a form of smokeless tobacco made from raw tobacco leaves and flavouring essence such as lime and saffron.

mospi.nic.in/sample-surveys

NSS 58’s data dictionary contains errors that prevented us from appropriately reading the data files; and, NSS 65, 67, 69-71 do not have household consumer expenditure components.

stats.oecd.org

population.un.org/wpp

We do not report 95% confidence intervals when the number of observations is less than 5.
