# Supplementary material for: Visualizing data: Trends in smoking tobacco prices and taxes in India
Source: Gates Open Res. Author manuscript. (PMC6367560; doi:10.12688/gatesopenres.12894.1)
Supplement: Peer review history

# Peer review: Referee response for version 1

**DOI:** 10.21956/gatesopenres.13990.r26876

**Published:** 2019-02-06

**Authors:** Laura Rossouw

**Affiliations:** 1 University of Cape Town, Cape Town, South Africa

The article provides a visual summary of tobacco tax and price trends in India over two decades. More specifically, it evaluates these trends within the broader framework of movements in income, thereby assessing the impact on the affordability of tobacco. The research question is addressed using (predominantly) publicly available, routinely collected data sources. The article is technically rigorous: the data analysis is robust, the conclusions well-summarised, and the article is thorough and well-written.

 My only major comment on the article is that, while it is technically sound, the findings could be strengthened by restructuring the research objective. While a descriptive analysis of tobacco price and tax trends in a specific country is interesting, the article plays a more important role in (1) illustrating the usefulness of using routinely collected government data and (2) acting as a rationale for open data access and perhaps even data storage houses. This is not completely clear in the article’s current introduction, which jumps around conceptually. The real objective of the paper is better summarised in the final paragraph of the conclusion, namely:

 “In sum, we establish the feasibility of using routinely collected tax and price data from national consumer price surveys to evaluate tobacco control policies. The CPIs are collected widely and offer a low-cost, generally publicly available dataset to track tobacco taxation. In India, these routine data reveal that the sub-optimal use of large increases in excise taxes are not changing affordability of cigarettes in particular.”

 Addressing the research question within this broader framework of using existing data to address tax-related policy questions, and promoting public access to government data sources, the authors could consider addressing other relevant questions. What are the challenges and opportunities for making government data more freely available – what is the feasibility of this in LMICs? While open-access government data promotes transparency and accountability, are there risks of misuse? What about inaccurate data – is there any way of checking this data quality? How can we determine that government data is correct? What are the risks associated with open access government data? Are there questions of ethics and responsibility when re-using government data? If government data is used more liberally for different purposes, will data quality issues result in the slower release of government data?

 There are also some smaller points for the authors to consider:

- Page 3: “Recently, there has been calls for economists and public policy practitioners to make better use of data visualization” – up until this point, the rationale for the article has focused on data collection – data vizualisation seems out of place here.
- Page 3: “where decades of good life are lost compared to otherwise similar non-smokers”. Should this be “quality life”?
- Page 3: “Cigarettes that are no more than 75 mm”- should be ‘less than’ rather than ‘no more than’.
- Methodology section: Is the routinely collected data being used in this analysis available in other LMICs too?
- Table 1 – just fix strange breadth of columns.
- Page 12, sentence “When rising income is taken into account, both cigarettes and bidis have not become less affordable between 2000 and 2018”. I understand that affordability is a difficult concept to describe, but can one write this in a more intuitive way? For instance, “affordability has remained unchanged over this period”, or even become more affordable.

I have read this submission. I believe that I have an appropriate level of expertise to confirm that it is of an acceptable scientific standard.

# Peer review: Referee response for version 1

**DOI:** 10.21956/gatesopenres.13990.r26873

**Published:** 2019-02-05

**Authors:** Shreelata Rao Seshadri

**Affiliations:** 1 Azim Premji University, Bengaluru, India

**General Comments**:

 The paper deals with a critical public health issue, using data not commonly utilized to study trends in tobacco prices and taxes. The visualization of the data is interesting, and makes it accessible to the general reader. The paper contributes importantly to evidence that supports decision-making on tobacco taxation policies, an area that needs a lot more attention in India. It also highlights the inter-state variation in the application of taxation as a tool to influence smoking tobacco consumption. However, while arguing for higher taxes, the paper makes no mention of the impact of this policy decision on (i) consumption levels and decisions to quit, (ii) uptake of alternative tobacco products, and (iii) tobacco consumption choices across different demographic strata. Such issues complicate the impact of tobacco control measures, and an acknowledgement of this complexity would have added richness to the analysis.   

**Specific comments**:

1. When mentioning the Framework Convention on Tobacco Control in the Introduction, the authors may also mention that India has adopted the Cigarettes and Other Tobacco Products Act (COTPA, 2003), which does not deal with tobacco taxation, but does make important provisions against smoking in public places, as well as advertisement and sales of smoking tobacco. These have also been found to be effective tobacco control interventions (DCP3), although tobacco taxation is potentially the most effective. Particularly when price increases threaten sales, the aggressive marketing of tobacco products in new geographies and demographies (particularly among adolescents) is a serious possibility. In such a circumstance, provisions against promotion and advertising and in favor of information campaigns have a positive impact.
2. The paper shows an increase in affordability of cigarettes and bidis by comparing the Consumer Price Index for tobacco to GDP per capita. The implication here is that greater affordability will lead to greater tobacco consumption. Yet recent survey data show (National Family Health Survey Rounds 3 and 4 - http://rchiips.org/nfhs/factsheet_nfhs-4.shtml) that tobacco consumption among men 15-49 years of age in India overall, and in both Rajasthan and Uttar Pradesh have reduced between 2005/06 and 2015/16: from 57.0 to 44.5% in India, 60.4 to 46.9% in Rajasthan and from 64.3 to 53.0% in Uttar Pradesh. Also, a large proportion of men in this age group have tried to quit tobacco in the 12 months preceding the survey: 30.6%, 25.8% and 38.7% for India, Rajasthan and Uttar Pradesh respectively. This seems to indicate that something other than price and affordability are driving consumption behavior.
3. The impact of increasing cigarette and bidi taxes on switching to alternative tobacco products needs to be mentioned. Consumption of non-smoking tobacco exceeds consumption of smoking tobacco across the board in India. For example, 34.2% of men aged 15-49 in Rajasthan consume ghutka and khaini as compared to 26.5% who consume cigarettes and bidis. The same is true in Uttar Pradesh: 45.7% consuming ghutka and khaini versus 28.4% consuming cigarettes and bidis. If taxes are raised to the point where cigarettes and bidis become unaffordable, a recommendation of the paper, what does the evidence say about the adoption of alternative tobacco products instead? Given the lethal impact of chewing tobacco in terms of oral and other cancers, the policy implications may not be linear.
4. The above point is particularly critical for those groups whose tobacco product of choice is the chewing variety, such as women. Three times as many women chew tobacco with ghutka or paan masala as smoke bidis in Rajasthan (3.6% vs 1.3%) and four times as many in Uttar Pradesh (4% vs 1%). The impact of taxation on smoking tobacco would be minimal in this context.

I have read this submission. I believe that I have an appropriate level of expertise to confirm that it is of an acceptable scientific standard.

# Peer review: Referee response for version 1

**DOI:** 10.21956/gatesopenres.13990.r26875

**Published:** 2019-01-31

**Authors:** Rijo M. John

**Affiliations:** 1 Centre for Public Policy Research (CPPR), Kochi, India

**General Comments:**

 This paper is an excellent repository of data on trends in prices and taxes of bidis and cigarettes in India over a long period. The authors have meticulously collated this data from different secondary sources which are usually difficult to access for most people and putting them together in one place. This can be an important reference for researchers, policy makers and civil society organizations. Hence, it is important that the presented data is fully accurate. However, there are few places where the paper needs revision to make the presented data fully accurate and consistent with the available secondary sources.

**Specific Comments:**

1. Introduction 2 Paragraph: Please clarify that not only the Basic Excise Duty (BED), but other taxes such as National Calamity Contingent Duty (NCCD) and Additional Duties of Excise (health cess) applied on cigarettes also vary by length and filter.
2. Introduction 2 Paragraph: The article says “in June 2018, the specific cigarette tax on the most popular filter cigarettes (> 60 to 70 mm) was approximately 28 Rupees”. Please note that the cigarette tiers for the purpose of taxation is defined for <65 mm, 65-70mm, 70-75mm and 75mm & above. The excise tax varied for each of these tiers. It is not clear where the Rs.28 came from or which tax tier it refers to. Moreover, the Goods and Services Tax (GST) was introduced in June 2017. Which means in June 2018 it is the GST that is in place and the specific tax the article refers to is part of what is called a compensation cess under the GST. Because the authors discuss about specific excise taxes on cigarettes in the same paragraph, the readers may inadvertently believe this Rs. 28 is also a specific excise tax when it is actually not.
3. Introduction 2 Paragraph: Article says “all State VATs were repealed and replaced by a national Goods and Services Tax (GST) “ – Please clarify not only the VAT but other taxes like BED, health cess, bidi workers welfare cess etc. were also subsumed into GST except NCCD.
4. Method--self-reported unit values: The 58 round of NSS also collected data on consumer expenditures and it is not clear why this particular round of data was not included in the analysis in the paper.
5. Table 1 last row: the values specified against the year 2017/18 are not central excise figures unlike what the table heading suggests. GST on cigarettes now consists of a standard GST rate (28%), a compensation cess that has both ad valorem and specific components (both vary by length of cigarettes) and NCCD (varying by length of cigarettes). The values presented in the last row are the sum of compensation cess—specific part—under the GST and the NCCD. These are not excise taxes as the excise taxes are subsumed into the GST. The additional 5% and 36% applied are also part of the compensation cess under the GST and not excise.
6. Figure 1: This figure should make it clear whether there has been any real tax increase on bidis during the same time period. Whereas the figure shows major tax policy changes for cigarettes, such information is missing for bidis. Yet, we see that the series for both bidis and cigarettes moves in more or less the same direction. Has the bidi prices kept up with cigarette prices despite not having any tax increases during this period or has there been any significant tax increases on bidis corresponding to tax increases on cigarettes during this time? This is an information the authors should provide.
7. The title of figure 1 says there are three series; CPI-IW for bidis, cigarettes and all-items. However, the figure shows the series only for bidis and cigarettes.
8. The footnote to Figure 1 says “National GST introduced (28%); all state-VATs repealed;, 21-24% real tax increase on cigarettes”: First of all, not only the state –VATs but also the central excise was subsumed in GST. Secondly, it is incorrect to say there has been about 21-24% real tax increase on cigarettes post GST. The GST council had the goal of making tax rates largely revenue neutral post GST and the effective difference in tax burden between pre and post GST on cigarettes, and to an extend on bidis, were largely negligible. A recent paper which specifically examined the impact of GST on taxation of tobacco products in India ^1^ concluded that tax burden increased by only 0.14 percentage points post GST on cigarettes. A simple comparison of absolute rates prevailed prior to GST and post GST may inadvertently imply a significant increase in tax. However, pre-GST, the VAT was computed on the excise inclusive price whereas, under the GST, such tax on tax is fully done away with. As a result, the effective tax burden remains more or less the same for cigarettes before and after GST.
9. Figures 3a & 3b: From the financial year 2013-14 VAT rates on cigarettes and bidis went up to 65% from the previous year’s 50% in Rajasthan and not 60% as shown in these figures. Moreover, from the financial year 2014-15 onwards, Rajasthan introduced specific taxation instead of ad valorem taxes, for cigarettes. The authors have, however, not indicated this anywhere on the graph.
10. Figures 4a & 4b: From the financial year 2012-13 VAT rates on cigarettes went up to 55% (including a 5% additional tax) from the previous year’s 17.5% in Uttar Pradesh and it decreased to 30% (including 5% additional tax) in the subsequent year. It again went up to 45% in FY 2015-16. For bidis, however, such VAT changes have not happened during this time. It remained at 13.5% throughout this period. The authors should correct the figures accordingly.

I have read this submission. I believe that I have an appropriate level of expertise to confirm that it is of an acceptable scientific standard, however I have significant reservations, as outlined above.

## References

[1] JohnRMDauchyEGoodchildM: Estimated impact of the GST on tobacco products in India. *Tob Control*.2018; 10.1136/tobaccocontrol-2018-054479 10.1136/tobaccocontrol-2018-054479 30219796
